# Local unsupervised learning rules for a spiking neural network with dendrite

**DOI:** 10.1186/1471-2202-12-S1-P210

**Published:** 2011-07-18

**Authors:** Olivier FL Manette

**Affiliations:** 1 Universidad Nacional de Colombia, Colombia

## 

How could synapse number and position on a dendrite affect neuronal behavior with respect to the decoding of firing rate and temporal pattern? We developed a model of a neuron with a passive dendrite and found that dendritic length and the particular synapse positions directly determine the behavior of the neuron in response to patterns of received inputs. We revealed two distinct types of behavior by simply modifying the position and the number of synapses on the dendrite. In one setting – spatio-temporally sensitive - the neuron responds to a precise spatio-temporal pattern of spikes, but shows little change following an increase in the average frequency of the same input pattern. In the other setting – frequency sensitive - the neuron is insensitive to the precise arrival time of each spike but responds to changes in the average firing rate. This would allow neurons to detect different spatio-temporal patterns.

## Learning of spiking neurons with a dendrite

We present four local rules to train a network of spiking dendritic neurons.

**Figure 1:**
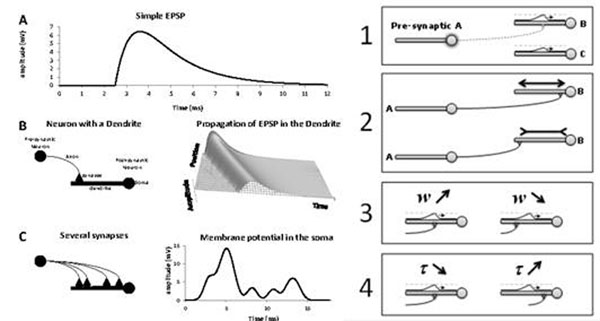
**Left Panel:A.** EPSP generated with equation 1, with the weight w=6mV, the firing time t_f_=2ms, the transmission delay Δ_ax_=0.5ms, the rising time constant: 1ms, and the time constant: t=2ms. **B**. Effect of a synapse being located on a dendrite rather than directly on a soma: it propagates as a function of its position in relation to or distance from the soma. **C.** Several EPSPs originating from different synapses at different firing times are combined to create a complex membrane potential in the soma as a function of time. **Right Panel: 1**. Creation of a new connection between two neurons: when a neuron is activated – here neuron A – it will try to make a new connection on the most depolarized neuron of its pool – here neuron B. The local membrane potentials of the dendrites are represented by a small graph above each dendrite. Here we see that neuron B is locally activated where the future synapse will be formed. While neuron C is also locally activated, the associated membrane potential does not reach the local threshold. **2**. Changing the length of the dendrite: If the last synapse is created very close to the soma then it will increase the length of the dendrite. If no synapse is connected on a free space on the dendrite between the last synapse and the soma, then the dendrite will shrink. **3**. Modification of synaptic weights: If a synapse is activated while the local membrane potential at the position of the synapse is high and reaches a local threshold, then, the weight is increased (*coactivation*). If the synapse is activated *after* the local membrane potential was high then it will decrease its weight. **4**. Changing the time constants of EPSP: if there is coactivation of the synapse and the local membrane potential at the position of the synapse then the time constant will be reduced. If the synapse is activated *before* the activation

After training, every neuron of the network becomes specialized for a particular feature of the input signal. With these rules, the network acts as a features extractor where each neuron contains a TAND vector, similar to logical AND but including information about time between the two events in the input signal. Figure 1.

